# Aqua­glutarato(2,4,6-tri-2-pyridyl-1,3,5-triazine)nickel(II) trihydrate

**DOI:** 10.1107/S1600536811006842

**Published:** 2011-03-02

**Authors:** Chun-Hua Jin

**Affiliations:** a Key Laboratory of Applied Marine Biotechnology, Ministry of Education, Ningbo University, Ningbo 315211, People’s Republic of China

## Abstract

In the title compound, [Ni(C_5_H_6_O_4_)(C_18_H_12_N_6_)(H_2_O)_2_]·3H_2_O, the Ni^II^ atom shows a distorted octa­hedral coordination by three N atoms of the tridentate chelating ligand and three O atoms of two aqua ligands and an O atom of one carboxylate group of the glutarate anion. Mol­ecules are self-assembled *via* inter­molecular O—H⋯O and O—H⋯N hydrogen-bonding inter­actions and π–π stacking inter­actions [centroid–centroid distance = 3.836 (3) Å] into a supra­molecular network.

## Related literature

For general background to 2,4,6-tris­(2-pyrid­yl)-1,3,5-triazine (tptz), see: Glaser *et al.* (2004 ▶); Zibaseresht & Hartshorn (2005 ▶); Zheng *et al.* (2006 ▶); Zhou *et al.* (2007 ▶). For potential applications of tptz-containing complexes, see: Gupta *et al.* (1993 ▶); Witter & Luther (2002 ▶).
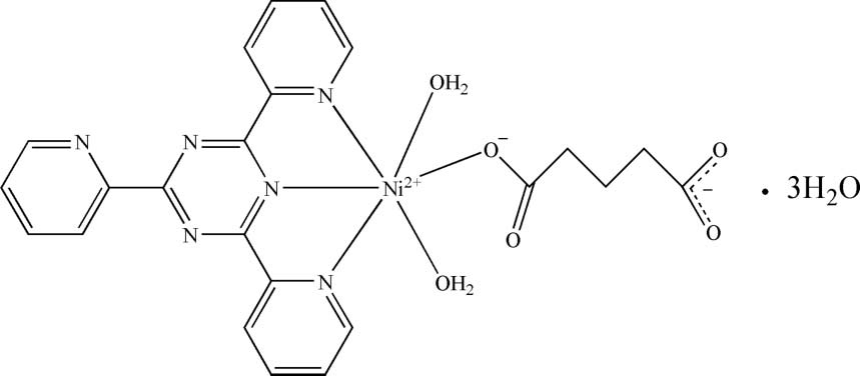


## Experimental

### 

#### Crystal data

[Ni(C_5_H_6_O_4_)(C_18_H_12_N_6_)(H_2_O)_2_]·3H_2_O
*M* *_r_* = 591.22
Triclinic, 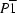

*a* = 9.3437 (19) Å
*b* = 10.486 (2) Å
*c* = 14.320 (3) Å
α = 83.09 (3)°
β = 87.16 (3)°
γ = 69.02 (3)°
*V* = 1300.5 (5) Å^3^
*Z* = 2
Mo *K*α radiation
μ = 0.81 mm^−1^
*T* = 295 K
0.30 × 0.23 × 0.17 mm

#### Data collection

Rigaku R-AXIS RAPID diffractometer
Absorption correction: multi-scan (*ABSCOR*; Higashi, 1995 ▶) *T*_min_ = 0.796, *T*_max_ = 0.868
12866 measured reflections
5901 independent reflections
4575 reflections with *I* > 2σ(*I*)
*R*_int_ = 0.023

#### Refinement

*R*[*F* ^2^ > 2σ(*F* ^2^)] = 0.030
*wR*(*F* ^2^) = 0.087
*S* = 1.14
5901 reflections
382 parameters
H atoms treated by a mixture of independent and constrained refinement
Δρ_max_ = 0.40 e Å^−3^
Δρ_min_ = −0.38 e Å^−3^

### 

Data collection: *RAPID-AUTO* (Rigaku, 1998 ▶); cell refinement: *RAPID-AUTO*; data reduction: *CrystalStructure* (Rigaku/MSC, 2004 ▶); program(s) used to solve structure: *SHELXS97* (Sheldrick, 2008 ▶); program(s) used to refine structure: *SHELXL97* (Sheldrick, 2008 ▶); molecular graphics: *XP* in *SHELXTL* (Sheldrick, 2008 ▶); software used to prepare material for publication: *SHELXL97*.

## Supplementary Material

Crystal structure: contains datablocks global, I. DOI: 10.1107/S1600536811006842/pk2302sup1.cif

Structure factors: contains datablocks I. DOI: 10.1107/S1600536811006842/pk2302Isup2.hkl

Additional supplementary materials:  crystallographic information; 3D view; checkCIF report

## Figures and Tables

**Table 1:** Hydrogen-bond geometry (Å, °)

*D*—H⋯*A*	*D*—H	H⋯*A*	*D*⋯*A*	*D*—H⋯*A*
O5—H5*B*⋯O8	0.77 (3)	1.97 (3)	2.719 (3)	167 (3)
O5—H5*C*⋯O4^i^	0.87 (3)	1.79 (3)	2.651 (2)	176 (4)
O6—H6*B*⋯O3^i^	0.86 (2)	1.80 (3)	2.655 (2)	173 (3)
O6—H6*C*⋯O3^ii^	0.82 (3)	1.90 (3)	2.706 (3)	166 (3)
O7—H7*B*⋯O4^i^	0.82 (3)	2.12 (3)	2.940 (3)	172 (3)
O7—H7*C*⋯O2^i^	0.86 (4)	1.91 (4)	2.747 (3)	166 (3)
O8—H8*B*⋯O9^iii^	0.82 (4)	1.92 (4)	2.695 (3)	157 (4)
O8—H8*C*⋯O7	0.84 (4)	1.96 (4)	2.753 (3)	156 (4)
O9—H9*B*⋯O8	0.86 (4)	2.06 (4)	2.907 (3)	170 (4)
O9—H9*C*⋯N6^iv^	0.75 (4)	2.18 (4)	2.854 (3)	150 (4)

Symmetry codes: (i) 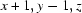; (ii) 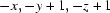; (iii) 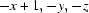; (iv) 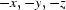.

**Fig. 1.**
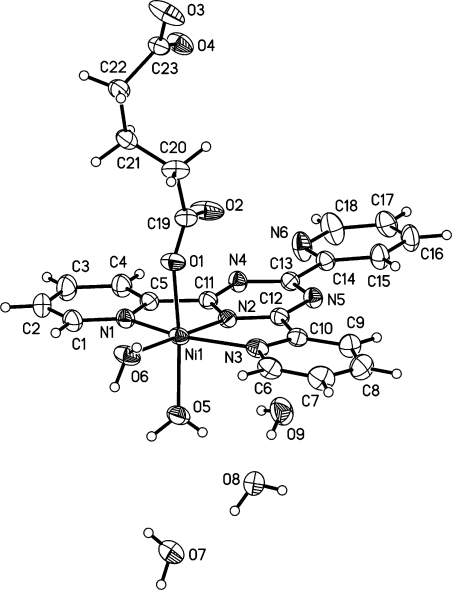
The asymmetric unit of the title compound, with the atom-labelling scheme. Displacement ellisoids are drawn at the 45% probability level.

## supplementary crystallographic information

### Comment

As an interesting polydentate nitrogen donor ligand, 2,4,6-tris(2-pyridyl)-1,3,5-triazine (tptz) has attracted increasing attention in the synthesis of novel transition metal complexes (Glaser, *et al.*, 2004; Zibaseresht & Hartshorn, 2005; Zheng, *et al.*, 2006; Zhou, *et al.*, 2007). It was applied in the extraction and separation of metal ions, and in the preparation of DNA cleaving agents. It has also been widely empolyed to determine the concentration of mono- and polysaccharides in seawater (Gupta, *et al.*, 1993; Witter & Luther, *et al.*, 2002). Our interest in tptz transition metal complexes prompts us to report a new tptz containing structure, [Ni(C_18_H_12_N_6_)(C_5_H_6_O_4_)(H_2_O)_2_].3H_2_O, obtained by self-assembly from Ni^II^, tptz and glutaric acid in aqueous methanolic solution.

The title compound, consists of [Ni(C_18_H_12_N_6_)(C_5_H_6_O_4_)(H_2_O)_2_] complex molecules and three lattice waters, as shown in Fig. 1, in which the Ni atoms are in a distorted octahedral coordination environment defined by three N atoms of one tridentate tptz ligand and three O atoms of two aqua ligands and the glutarate group. Three nitrogen atoms (N1, N2 and N3) from the tptz ligand and one O atom (O6) of water molecule form the equatorial base, while the carboxylate O atom (O1) and the other water ligand O atom (O5) occupy the axial positions. Ni-N distances vary from 1.986 (2) to 2.173 (2) Å, and the Ni-O bond distances fall in the region 2.021 (2) to 2.056 (2) Å. The deviation from the ideal octahedral geometry is indicated by the difference in *cisoid* [76.38 (7)°-106.06 (7)°] and *transoid* angles [153.59 (6)°-177.01 (7)°]. In the tptz ligand, the C(sp^2^)-C(sp^2^) distances within the ring are normal [1.373 (4)-1.392 (2) Å], and the exterior bond distances, 1.482 (3)-1.491 (3) Å, are also normal. The tptz ligands deviate little from planarity, the three pyridyl rings are twisted with respect to the central triazine ring by angles of 3.4 (2)°, 4.1 (1)° and 8.6 (1)° with the N3 ring displaying the highest degree of twisting. The [Ni(C_18_H_12_N_6_)(C_5_H_6_O_4_)(H_2_O)_2_] complex molecules are assembled into a 3D network by hydrogen bonds between the coordinated water and lattice water molecules and the carboxylate group and pyridyl nitrogen atoms with the distances from 2.651 (4) Å to 2.938 (5) Å (Table 1). The crystal structure is also stabilized by intermolecular π-π stacking interactions between tptz ligands [centroid-centroid distance = 3.836 (3) Å].

### Experimental

Dropwise addition of 2.0 ml NaOH (1.0 M) to a stirred solution of NiCl_2_.6H_2_O (0.238 g, 1.00 mmol) in 5.0 ml H_2_O produced a green precipitate, which was then centrifuged and washed with double-distilled water until no Cl^-^ anions were detectable. The collected precipitate and 2,4,6-tri(2-pyridyl)-1,3,5-triazine (tptz) (0.312 g, 1.00 mmol) were added to a stirred solution of glutaric acid (0.132 g, 1.00 mmol) in CH_3_OH/H_2_O (30.0 ml; 1:1 v/v). The resulting mixture was stirred for a further 30 min. After filtration, the dark green filtrate (pH = 5.12) was evaporated slowly at room temperature and afforded dark green crystals over two weeks (yield 45.4%, based on initial NiCl_2_ input).

### Refinement

All H atoms bound to C were position geometrically and refined as riding, with C-H = 0.93 Å and *U*_iso_(H) = 1.2*U*_eq_(C). H atoms attached to O atoms were found in a difference Fourier map and refined freely with *U*_iso_(H) = 1.5*U*_eq_(O).

### Crystal data

**Table:**

[Ni(C_5_H_6_O_4_)(C_18_H_12_N_6_)(H_2_O)_2_]·3H_2_O	*Z* = 2
*M**_r_* = 591.22	*F*(000) = 616
Triclinic, *P*1	*D*_x_ = 1.510 Mg m^−^^3^
Hall symbol: -P 1	Mo *K*α radiation, λ = 0.71073 Å
*a* = 9.3437 (19) Å	Cell parameters from 10176 reflections
*b* = 10.486 (2) Å	θ = 3.0–27.5°
*c* = 14.320 (3) Å	µ = 0.81 mm^−^^1^
α = 83.09 (3)°	*T* = 295 K
β = 87.16 (3)°	Block, dark green
γ = 69.02 (3)°	0.30 × 0.23 × 0.17 mm
*V* = 1300.5 (5) Å^3^	

### Data collection

**Table:**

Rigaku R-AXIS RAPID diffractometer	5901 independent reflections
Radiation source: fine-focus sealed tube	4575 reflections with *I* > 2σ(*I*)
graphite	*R*_int_ = 0.023
ω scans	θ_max_ = 27.5°, θ_min_ = 3.0°
Absorption correction: multi-scan (*ABSCOR*; Higashi, 1995)	*h* = −12→11
*T*_min_ = 0.796, *T*_max_ = 0.868	*k* = −13→13
12866 measured reflections	*l* = −18→18

### Refinement

**Table:**

Refinement on *F*^2^	Primary atom site location: structure-invariant direct methods
Least-squares matrix: full	Secondary atom site location: difference Fourier map
*R*[*F*^2^ > 2σ(*F*^2^)] = 0.030	Hydrogen site location: inferred from neighbouring sites
*wR*(*F*^2^) = 0.087	H atoms treated by a mixture of independent and constrained refinement
*S* = 1.14	*w* = 1/[σ^2^(*F*_o_^2^) + (0.0352*P*)^2^ + 0.3875*P*] where *P* = (*F*_o_^2^ + 2*F*_c_^2^)/3
5901 reflections	(Δ/σ)_max_ < 0.001
382 parameters	Δρ_max_ = 0.40 e Å^−^^3^
0 restraints	Δρ_min_ = −0.38 e Å^−^^3^

### Special details

**Table:**

Geometry. All esds (except the esd in the dihedral angle between two l.s. planes) are estimated using the full covariance matrix. The cell esds are taken into account individually in the estimation of esds in distances, angles and torsion angles; correlations between esds in cell parameters are only used when they are defined by crystal symmetry. An approximate (isotropic) treatment of cell esds is used for estimating esds involving l.s. planes.
Refinement. Refinement of F^2^ against ALL reflections. The weighted R-factor wR and goodness of fit S are based on F^2^, conventional R-factors R are based on F, with F set to zero for negative F^2^. The threshold expression of F^2^ > 2σ(F^2^) is used only for calculating R-factors(gt) etc. and is not relevant to the choice of reflections for refinement. R-factors based on F^2^ are statistically about twice as large as those based on F, and R- factors based on ALL data will be even larger.

### Fractional atomic coordinates and isotropic or equivalent isotropic displacement parameters (Å^2^)

**Table:**

	*x*	*y*	*z*	*U*_iso_*/*U*_eq_	
Ni1	0.23523 (3)	0.10184 (3)	0.306650 (17)	0.02922 (8)	
N1	0.07072 (19)	0.00148 (16)	0.31671 (11)	0.0314 (4)	
N2	0.14608 (18)	0.14756 (16)	0.17850 (11)	0.0279 (3)	
N3	0.36324 (19)	0.21923 (17)	0.23120 (12)	0.0338 (4)	
N4	−0.05102 (19)	0.15524 (17)	0.08066 (11)	0.0317 (4)	
N5	0.11453 (18)	0.28123 (17)	0.03247 (11)	0.0318 (4)	
N6	−0.1961 (2)	0.2413 (2)	−0.08469 (13)	0.0474 (5)	
C1	0.0402 (3)	−0.0764 (2)	0.39029 (15)	0.0399 (5)	
H1A	0.0992	−0.0955	0.4441	0.048*	
C2	−0.0753 (3)	−0.1299 (2)	0.38989 (16)	0.0465 (6)	
H2A	−0.0936	−0.1833	0.4425	0.056*	
C3	−0.1624 (3)	−0.1029 (3)	0.31043 (17)	0.0495 (6)	
H3A	−0.2409	−0.1374	0.3087	0.059*	
C4	−0.1320 (2)	−0.0233 (2)	0.23247 (16)	0.0400 (5)	
H4A	−0.1888	−0.0043	0.1777	0.048*	
C5	−0.0149 (2)	0.0266 (2)	0.23880 (13)	0.0299 (4)	
C6	0.4845 (2)	0.2426 (2)	0.26107 (17)	0.0409 (5)	
H6A	0.5158	0.2128	0.3231	0.049*	
C7	0.5645 (3)	0.3090 (2)	0.20348 (18)	0.0481 (6)	
H7A	0.6487	0.3228	0.2263	0.058*	
C8	0.5187 (3)	0.3547 (3)	0.11206 (19)	0.0494 (6)	
H8A	0.5718	0.3994	0.0723	0.059*	
C9	0.3922 (3)	0.3334 (2)	0.07943 (16)	0.0407 (5)	
H9A	0.3579	0.3643	0.0181	0.049*	
C10	0.3190 (2)	0.2646 (2)	0.14127 (14)	0.0309 (4)	
C11	0.0268 (2)	0.11409 (19)	0.16027 (13)	0.0282 (4)	
C12	0.1862 (2)	0.23092 (19)	0.11369 (13)	0.0287 (4)	
C13	−0.0020 (2)	0.2389 (2)	0.01894 (13)	0.0297 (4)	
C14	−0.0849 (2)	0.2889 (2)	−0.07149 (14)	0.0305 (4)	
C15	−0.0487 (3)	0.3795 (2)	−0.13838 (15)	0.0416 (5)	
H15A	0.0281	0.4127	−0.1275	0.050*	
C16	−0.1293 (3)	0.4197 (3)	−0.22184 (16)	0.0487 (6)	
H16A	−0.1067	0.4802	−0.2680	0.058*	
C17	−0.2416 (3)	0.3701 (2)	−0.23610 (16)	0.0464 (6)	
H17A	−0.2965	0.3952	−0.2919	0.056*	
C18	−0.2713 (3)	0.2819 (3)	−0.16543 (18)	0.0569 (7)	
H18A	−0.3487	0.2486	−0.1748	0.068*	
O1	0.08747 (16)	0.26850 (15)	0.36329 (10)	0.0393 (3)	
O2	−0.10065 (19)	0.35714 (17)	0.25867 (13)	0.0557 (5)	
O3	−0.4597 (2)	0.80336 (16)	0.48537 (14)	0.0665 (6)	
O4	−0.48137 (19)	0.68559 (15)	0.37304 (11)	0.0473 (4)	
C19	−0.0383 (2)	0.3554 (2)	0.33396 (15)	0.0348 (4)	
C20	−0.1175 (2)	0.4682 (2)	0.39654 (17)	0.0432 (5)	
H20A	−0.0432	0.4733	0.4395	0.052*	
H20B	−0.1562	0.5556	0.3577	0.052*	
C21	−0.2492 (3)	0.4441 (2)	0.45288 (17)	0.0451 (5)	
H21A	−0.2093	0.3605	0.4956	0.054*	
H21B	−0.3194	0.4314	0.4103	0.054*	
C22	−0.3360 (3)	0.5631 (2)	0.50912 (15)	0.0426 (5)	
H22A	−0.4034	0.5345	0.5536	0.051*	
H22B	−0.2628	0.5831	0.5451	0.051*	
C23	−0.4307 (2)	0.6938 (2)	0.45040 (15)	0.0353 (4)	
O5	0.4055 (2)	−0.07320 (18)	0.27026 (12)	0.0506 (4)	
H5B	0.456 (4)	−0.079 (3)	0.226 (2)	0.076*	
H5C	0.443 (4)	−0.150 (3)	0.306 (2)	0.076*	
O6	0.32425 (19)	0.04577 (17)	0.43739 (11)	0.0427 (4)	
H6B	0.394 (3)	−0.034 (3)	0.448 (2)	0.064*	
H6C	0.352 (3)	0.103 (3)	0.458 (2)	0.064*	
O7	0.6705 (2)	−0.4023 (2)	0.19485 (15)	0.0600 (5)	
H7B	0.625 (4)	−0.384 (4)	0.245 (2)	0.090*	
H7C	0.747 (4)	−0.478 (4)	0.205 (2)	0.090*	
O8	0.5802 (2)	−0.1346 (2)	0.11286 (14)	0.0567 (5)	
H8B	0.636 (4)	−0.095 (4)	0.088 (2)	0.085*	
H8C	0.633 (4)	−0.216 (4)	0.133 (2)	0.085*	
O9	0.3067 (2)	−0.03481 (19)	−0.00273 (15)	0.0595 (5)	
H9B	0.387 (4)	−0.074 (4)	0.031 (2)	0.089*	
H9C	0.262 (4)	−0.082 (4)	0.002 (3)	0.089*	

### Atomic displacement parameters (Å^2^)

**Table:**

	*U*^11^	*U*^22^	*U*^33^	*U*^12^	*U*^13^	*U*^23^
Ni1	0.02710 (14)	0.02892 (13)	0.02707 (14)	−0.00368 (10)	−0.00675 (10)	−0.00274 (9)
N1	0.0344 (9)	0.0315 (8)	0.0248 (8)	−0.0081 (7)	−0.0018 (7)	−0.0008 (7)
N2	0.0265 (8)	0.0294 (8)	0.0266 (8)	−0.0086 (7)	−0.0031 (6)	−0.0017 (6)
N3	0.0284 (8)	0.0334 (9)	0.0377 (9)	−0.0076 (7)	−0.0082 (7)	−0.0046 (7)
N4	0.0321 (9)	0.0362 (9)	0.0275 (8)	−0.0137 (8)	−0.0061 (7)	0.0023 (7)
N5	0.0311 (9)	0.0347 (9)	0.0304 (8)	−0.0133 (7)	−0.0056 (7)	0.0011 (7)
N6	0.0540 (12)	0.0591 (12)	0.0383 (10)	−0.0351 (11)	−0.0199 (9)	0.0150 (9)
C1	0.0467 (13)	0.0385 (11)	0.0286 (10)	−0.0094 (10)	−0.0017 (9)	0.0016 (9)
C2	0.0496 (14)	0.0460 (13)	0.0390 (12)	−0.0158 (11)	0.0067 (10)	0.0076 (10)
C3	0.0443 (13)	0.0556 (14)	0.0518 (14)	−0.0263 (12)	−0.0007 (11)	0.0090 (12)
C4	0.0375 (11)	0.0441 (12)	0.0394 (12)	−0.0175 (10)	−0.0052 (9)	0.0037 (10)
C5	0.0289 (10)	0.0306 (10)	0.0276 (10)	−0.0077 (8)	−0.0027 (8)	−0.0005 (8)
C6	0.0345 (11)	0.0425 (12)	0.0457 (13)	−0.0124 (10)	−0.0143 (10)	−0.0029 (10)
C7	0.0360 (12)	0.0514 (14)	0.0621 (16)	−0.0201 (11)	−0.0143 (11)	−0.0064 (12)
C8	0.0426 (13)	0.0548 (14)	0.0588 (15)	−0.0276 (12)	0.0003 (11)	−0.0043 (12)
C9	0.0396 (12)	0.0449 (12)	0.0411 (12)	−0.0193 (10)	−0.0036 (10)	−0.0028 (10)
C10	0.0282 (10)	0.0300 (10)	0.0338 (10)	−0.0087 (8)	−0.0032 (8)	−0.0040 (8)
C11	0.0277 (9)	0.0276 (9)	0.0270 (9)	−0.0070 (8)	−0.0026 (8)	−0.0022 (7)
C12	0.0280 (10)	0.0279 (9)	0.0281 (9)	−0.0070 (8)	−0.0022 (8)	−0.0026 (8)
C13	0.0290 (10)	0.0308 (10)	0.0282 (10)	−0.0094 (8)	−0.0028 (8)	−0.0013 (8)
C14	0.0304 (10)	0.0312 (10)	0.0294 (10)	−0.0107 (8)	−0.0044 (8)	0.0001 (8)
C15	0.0427 (12)	0.0504 (13)	0.0353 (11)	−0.0240 (11)	−0.0077 (9)	0.0077 (10)
C16	0.0547 (14)	0.0554 (14)	0.0344 (12)	−0.0228 (12)	−0.0046 (10)	0.0139 (11)
C17	0.0525 (14)	0.0514 (13)	0.0328 (11)	−0.0175 (12)	−0.0172 (10)	0.0080 (10)
C18	0.0647 (16)	0.0673 (17)	0.0505 (14)	−0.0409 (15)	−0.0305 (13)	0.0161 (13)
O1	0.0332 (8)	0.0368 (8)	0.0388 (8)	0.0007 (6)	−0.0063 (6)	−0.0089 (6)
O2	0.0422 (9)	0.0523 (10)	0.0592 (11)	0.0077 (8)	−0.0209 (8)	−0.0240 (8)
O3	0.0843 (14)	0.0318 (8)	0.0698 (12)	0.0038 (9)	−0.0443 (11)	−0.0131 (8)
O4	0.0576 (10)	0.0363 (8)	0.0385 (9)	−0.0028 (7)	−0.0146 (7)	−0.0057 (7)
C19	0.0283 (10)	0.0339 (10)	0.0403 (11)	−0.0075 (9)	−0.0023 (9)	−0.0071 (9)
C20	0.0323 (11)	0.0402 (12)	0.0548 (14)	−0.0055 (10)	−0.0065 (10)	−0.0173 (11)
C21	0.0513 (14)	0.0279 (10)	0.0444 (13)	−0.0021 (10)	0.0034 (11)	0.0016 (9)
C22	0.0467 (13)	0.0358 (11)	0.0321 (11)	0.0000 (10)	0.0002 (9)	0.0000 (9)
C23	0.0342 (11)	0.0290 (10)	0.0366 (11)	−0.0035 (9)	−0.0044 (9)	−0.0035 (8)
O5	0.0563 (11)	0.0361 (8)	0.0383 (9)	0.0071 (8)	0.0062 (8)	0.0002 (7)
O6	0.0427 (9)	0.0372 (8)	0.0377 (8)	0.0011 (7)	−0.0176 (7)	−0.0052 (7)
O7	0.0537 (12)	0.0469 (10)	0.0649 (12)	−0.0047 (9)	−0.0044 (9)	0.0092 (10)
O8	0.0539 (11)	0.0508 (10)	0.0560 (11)	−0.0131 (9)	0.0140 (9)	0.0071 (9)
O9	0.0596 (12)	0.0499 (11)	0.0728 (13)	−0.0290 (10)	−0.0019 (10)	0.0093 (9)

### Geometric parameters (Å, °)

**Table:**

Ni1—N2	1.9862 (17)	C10—C12	1.488 (3)
Ni1—O6	2.0203 (16)	C13—C14	1.482 (3)
Ni1—O1	2.0300 (16)	C14—C15	1.383 (3)
Ni1—O5	2.0563 (19)	C15—C16	1.383 (3)
Ni1—N1	2.1426 (17)	C15—H15A	0.9300
Ni1—N3	2.1723 (18)	C16—C17	1.360 (3)
N1—C1	1.338 (3)	C16—H16A	0.9300
N1—C5	1.350 (2)	C17—C18	1.374 (3)
N2—C11	1.328 (2)	C17—H17A	0.9300
N2—C12	1.331 (2)	C18—H18A	0.9300
N3—C6	1.341 (3)	O1—C19	1.256 (2)
N3—C10	1.349 (3)	O2—C19	1.246 (3)
N4—C11	1.324 (2)	O3—C23	1.243 (3)
N4—C13	1.350 (2)	O4—C23	1.250 (3)
N5—C12	1.325 (2)	C19—C20	1.521 (3)
N5—C13	1.344 (2)	C20—C21	1.517 (3)
N6—C18	1.327 (3)	C20—H20A	0.9700
N6—C14	1.333 (3)	C20—H20B	0.9700
C1—C2	1.383 (3)	C21—C22	1.522 (3)
C1—H1A	0.9300	C21—H21A	0.9700
C2—C3	1.373 (3)	C21—H21B	0.9700
C2—H2A	0.9300	C22—C23	1.516 (3)
C3—C4	1.392 (3)	C22—H22A	0.9700
C3—H3A	0.9300	C22—H22B	0.9700
C4—C5	1.382 (3)	O5—H5B	0.76 (3)
C4—H4A	0.9300	O5—H5C	0.87 (3)
C5—C11	1.491 (3)	O6—H6B	0.86 (3)
C6—C7	1.378 (3)	O6—H6C	0.83 (3)
C6—H6A	0.9300	O7—H7B	0.82 (4)
C7—C8	1.373 (3)	O7—H7C	0.86 (4)
C7—H7A	0.9300	O8—H8B	0.82 (3)
C8—C9	1.391 (3)	O8—H8C	0.85 (4)
C8—H8A	0.9300	O9—H9B	0.86 (3)
C9—C10	1.382 (3)	O9—H9C	0.75 (3)
C9—H9A	0.9300		
			
N2—Ni1—O6	176.98 (7)	N4—C11—C5	122.12 (17)
N2—Ni1—O1	97.21 (7)	N2—C11—C5	113.91 (16)
O6—Ni1—O1	84.47 (7)	N5—C12—N2	123.69 (17)
N2—Ni1—O5	92.47 (8)	N5—C12—C10	122.62 (17)
O6—Ni1—O5	85.93 (8)	N2—C12—C10	113.68 (16)
O1—Ni1—O5	170.19 (7)	N5—C13—N4	125.81 (17)
N2—Ni1—N1	77.29 (7)	N5—C13—C14	117.32 (16)
O6—Ni1—N1	100.17 (7)	N4—C13—C14	116.87 (16)
O1—Ni1—N1	92.45 (6)	N6—C14—C15	121.97 (19)
O5—Ni1—N1	91.20 (8)	N6—C14—C13	116.15 (17)
N2—Ni1—N3	76.38 (7)	C15—C14—C13	121.88 (18)
O6—Ni1—N3	106.09 (7)	C16—C15—C14	118.6 (2)
O1—Ni1—N3	92.88 (7)	C16—C15—H15A	120.7
O5—Ni1—N3	87.86 (7)	C14—C15—H15A	120.7
N1—Ni1—N3	153.58 (6)	C17—C16—C15	119.7 (2)
C1—N1—C5	117.66 (17)	C17—C16—H16A	120.2
C1—N1—Ni1	128.33 (14)	C15—C16—H16A	120.2
C5—N1—Ni1	113.96 (12)	C16—C17—C18	117.9 (2)
C11—N2—C12	117.66 (16)	C16—C17—H17A	121.1
C11—N2—Ni1	120.20 (13)	C18—C17—H17A	121.1
C12—N2—Ni1	121.24 (13)	N6—C18—C17	123.9 (2)
C6—N3—C10	117.70 (18)	N6—C18—H18A	118.0
C6—N3—Ni1	128.03 (15)	C17—C18—H18A	118.0
C10—N3—Ni1	114.10 (13)	C19—O1—Ni1	130.93 (14)
C11—N4—C13	114.29 (16)	O2—C19—O1	125.7 (2)
C12—N5—C13	114.55 (16)	O2—C19—C20	118.44 (19)
C18—N6—C14	117.96 (18)	O1—C19—C20	115.89 (19)
N1—C1—C2	122.9 (2)	C21—C20—C19	112.55 (18)
N1—C1—H1A	118.5	C21—C20—H20A	109.1
C2—C1—H1A	118.5	C19—C20—H20A	109.1
C3—C2—C1	118.9 (2)	C21—C20—H20B	109.1
C3—C2—H2A	120.6	C19—C20—H20B	109.1
C1—C2—H2A	120.6	H20A—C20—H20B	107.8
C2—C3—C4	119.4 (2)	C20—C21—C22	112.16 (19)
C2—C3—H3A	120.3	C20—C21—H21A	109.2
C4—C3—H3A	120.3	C22—C21—H21A	109.2
C5—C4—C3	118.1 (2)	C20—C21—H21B	109.2
C5—C4—H4A	121.0	C22—C21—H21B	109.2
C3—C4—H4A	121.0	H21A—C21—H21B	107.9
N1—C5—C4	123.05 (18)	C23—C22—C21	114.68 (18)
N1—C5—C11	114.11 (16)	C23—C22—H22A	108.6
C4—C5—C11	122.83 (18)	C21—C22—H22A	108.6
N3—C6—C7	122.6 (2)	C23—C22—H22B	108.6
N3—C6—H6A	118.7	C21—C22—H22B	108.6
C7—C6—H6A	118.7	H22A—C22—H22B	107.6
C8—C7—C6	119.3 (2)	O3—C23—O4	123.91 (19)
C8—C7—H7A	120.3	O3—C23—C22	116.88 (19)
C6—C7—H7A	120.3	O4—C23—C22	119.10 (19)
C7—C8—C9	119.4 (2)	Ni1—O5—H5B	126 (3)
C7—C8—H8A	120.3	Ni1—O5—H5C	126 (2)
C9—C8—H8A	120.3	H5B—O5—H5C	107 (3)
C10—C9—C8	117.8 (2)	Ni1—O6—H6B	117 (2)
C10—C9—H9A	121.1	Ni1—O6—H6C	115 (2)
C8—C9—H9A	121.1	H6B—O6—H6C	109 (3)
N3—C10—C9	123.23 (18)	H7B—O7—H7C	109 (3)
N3—C10—C12	113.92 (17)	H8B—O8—H8C	110 (3)
C9—C10—C12	122.83 (18)	H9B—O9—H9C	107 (3)
N4—C11—N2	123.96 (17)		

### Hydrogen-bond geometry (Å, °)

**Table:**

*D*—H···*A*	*D*—H	H···*A*	*D*···*A*	*D*—H···*A*
O5—H5B···O8	0.77 (3)	1.97 (3)	2.719 (3)	167 (3)
O5—H5C···O4^i^	0.87 (3)	1.79 (3)	2.651 (2)	176 (4)
O6—H6B···O3^i^	0.86 (2)	1.80 (3)	2.655 (2)	173 (3)
O6—H6C···O3^ii^	0.82 (3)	1.90 (3)	2.706 (3)	166 (3)
O7—H7B···O4^i^	0.82 (3)	2.12 (3)	2.940 (3)	172 (3)
O7—H7C···O2^i^	0.86 (4)	1.91 (4)	2.747 (3)	166 (3)
O8—H8B···O9^iii^	0.82 (4)	1.92 (4)	2.695 (3)	157 (4)
O8—H8C···O7	0.84 (4)	1.96 (4)	2.753 (3)	156 (4)
O9—H9B···O8	0.86 (4)	2.06 (4)	2.907 (3)	170 (4)
O9—H9C···N6^iv^	0.75 (4)	2.18 (4)	2.854 (3)	150 (4)

Symmetry codes:  (i) *x*+1, *y*−1, *z*; (ii) −*x*, −*y*+1, −*z*+1; (iii) −*x*+1, −*y*, −*z*; (iv) −*x*, −*y*, −*z*.
